# Role of Sequence and Structural Polymorphism on the Mechanical Properties of Amyloid Fibrils

**DOI:** 10.1371/journal.pone.0088502

**Published:** 2014-02-14

**Authors:** Gwonchan Yoon, Myeongsang Lee, Jae In Kim, Sungsoo Na, Kilho Eom

**Affiliations:** 1 Department of Mechanical Engineering, Korea University, Seoul, Republic of Korea; 2 Department of Mechanical Engineering, Boston University, Boston, Massachusetts, United States of America; 3 Biomechanics Laboratory, College of Sport Science, Sungkyunkwan University, Suwon, Republic of Korea; University of Akron, United States of America

## Abstract

Amyloid fibrils playing a critical role in disease expression, have recently been found to exhibit the excellent mechanical properties such as elastic modulus in the order of 10 GPa, which is comparable to that of other mechanical proteins such as microtubule, actin filament, and spider silk. These remarkable mechanical properties of amyloid fibrils are correlated with their functional role in disease expression. This suggests the importance in understanding how these excellent mechanical properties are originated through self-assembly process that may depend on the amino acid sequence. However, the sequence-structure-property relationship of amyloid fibrils has not been fully understood yet. In this work, we characterize the mechanical properties of human islet amyloid polypeptide (hIAPP) fibrils with respect to their molecular structures as well as their amino acid sequence by using all-atom explicit water molecular dynamics (MD) simulation. The simulation result suggests that the remarkable bending rigidity of amyloid fibrils can be achieved through a specific self-aggregation pattern such as antiparallel stacking of β strands (peptide chain). Moreover, we have shown that a single point mutation of hIAPP chain constituting a hIAPP fibril significantly affects the thermodynamic stability of hIAPP fibril formed by parallel stacking of peptide chain, and that a single point mutation results in a significant change in the bending rigidity of hIAPP fibrils formed by antiparallel stacking of β strands. This clearly elucidates the role of amino acid sequence on not only the equilibrium conformations of amyloid fibrils but also their mechanical properties. Our study sheds light on sequence-structure-property relationships of amyloid fibrils, which suggests that the mechanical properties of amyloid fibrils are encoded in their sequence-dependent molecular architecture.

## Introduction

For last decades, it has been observed that denatured proteins are prone to form a self-assembled structure [1], particularly fibril structure referred to as “amyloid fibril” [2]–[4], which is ubiquitously found in patients suffering from various diseases ranging from neurodegenerative disease [5] to cardiovascular disease [6] and type II diabetes [7], [8]. For instance, islet amyloid polypeptide (IAPP) chains are aggregated to form an one-dimensional fibril structure, and such IAPP fibril has been found in patients suffering from type II diabetes [7]. This amyloid fibril in human pancreas is able to replace the β cell performing the insulin secretion in pancreas, which results in amyloid fibril-driven inhibition of insulin secretion leading to diabetes. In particular, amyloid fibril is able to disrupt the cell membrane leading to cellular apoptosis, which is attributed to the bending rigidity of amyloid fibril being higher than that of cell membrane [9].

Recently, it has been suggested that the mechanical behavior of amyloid fibrils has played a vital role on their biological functions such as disease expression [10]. For example, a recent study [11] has reported that the infectivity of prion disease is closely related to the fracture toughness of prion fibrils, since a fragmented prion fibril serves as an amyloid seed that results in the infectivity of prion disease [12]. Moreover, we have recently found that the disease-specific prion fibril exhibits higher elastic modulus than prion fibril that does not have the disease specificity [13]. Moreover, the size-dependent elastic modulus of disease-specific prion fibril provides an insight into the length scale of prion fibril that can act as a seed leading to prion infectivity [13]. These observations clearly demonstrate that the functional role of amyloid fibril on the disease expression may be highly correlated with the mechanical properties of amyloid fibrils.

In order to understand the mechanical properties of amyloid fibrils, experimental techniques such as atomic force microscope (AFM) experiment and computational simulations such as atomistic simulations have been widely utilized. Specifically, a recent study by Knowles, *et al.* [14] reports that amyloid fibrils, which are formed by self-aggregation of mechanically weak proteins, bear excellent mechanical properties such as elastic modulus of ∼10 GPa, which is comparable to that of other mechanical proteins such as actin filament [15], microtubule [16]–[18], and spider silk [19]–[21]. In recent studies [22], [23], it has been found that the mechanical properties of β-lactoglobulin amyloid fibrils are closely related to their molecular structural hierarchies, particularly the helical pitch of amyloid fibrils, their length, and thickness. This indicates that the mechanical properties of amyloid fibrils in a physiological condition may be encoded in their molecular architecture. Based on a coarse-grained model, Knowles and coworkers [14] suggested that the remarkable mechanical properties of amyloid fibrils are attributed to intermolecular forces between cross-β structures (i.e. β sheet layers). In particular, the mechanical properties of protein materials are determined from the intermolecular interactions between the building blocks (e.g. β sheet layer) of protein materials [24], [25]. Moreover, Buehler and colleagues [26] have also provided that, by using atomistic simulation, the geometric confinement of hydrogen bonds between β sheet layers results in the enhancement of the mechanical properties of β sheet-rich crystal, which is responsible for the remarkable mechanical strength of a spider silk. This suggests that intermolecular interactions (e.g. hydrogen bonds) between β sheet layers play a role as a chemical glue in the mechanical behavior of β sheet-rich protein materials. Furthermore, a recent study [27] has interestingly found that the mechanical properties (e.g. persistent length) of amyloid fibrils are dependent on their β sheet-richness. In particular, when β strands are replaced with α helices in amyloid fibril, the mechanical properties (persistent length) of amyloid fibril are degraded. This may imply that the remarkable mechanical properties of amyloid fibril are due to intermolecular interactions between β sheet layers. These observations suggest that the mechanical properties of amyloid fibrils may be encoded in their molecular architecture, which sheds light on the structure-property relationship of amyloid fibril.

Even though there have been a lot of recent attempts to characterize the mechanical properties of amyloid fibrils as described earlier, structure-property relationship of amyloid fibrils has not been fully understood. Nevertheless, there are recent efforts [28]–[31] that have been made to gain insight into how the polymorphic structures of amyloid fibrils are formed in a physiological condition. A recent study by Sawaya, *et al.* [28] has reported that the polymorphic structures of amyloid fibrils are ascribed to the patterns of chemical interactions between β sheet layers; this chemical interaction pattern is named as “steric zipper” pattern. In recent studies, it has been found that the thermodynamic stability of amyloid fibrils is determined from their steric zipper patterns [31] as well as their amino acid sequence [29], [30]. This indicates that the molecular architecture of amyloid fibrils is encoded in not only their steric zipper patterns but also their amino acid sequence. Moreover, in our previous study [32], we have provided that the mechanical properties of amyloid fibrils are related to their steric zipper patterns such that antiparallel stacking of β strands improves the bending rigidity of amyloid fibril. In addition, a recent study by Buehler and coworkers [26] has suggested that the mechanical properties of β sheet-rich protein materials are determined from stacking pattern of β strands. These studies indicate that the mechanical properties of β sheet-rich protein materials are highly correlated with intermolecular interaction patterns between β sheet layers. In other words, it is implied that the mechanical properties of β sheet-rich protein materials are determined from their detailed molecular architecture, which sheds light on structure-property relationship of amyloid fibrils. However, the sequence-structure-property relationship of amyloid fibrils has still remained elusive despite recent efforts [26], [32] that partially reveal the structure-property relationship of β sheet-rich protein materials such as amyloid fibril [32].

The characterization of sequence-structure-property relationships of protein materials such as amyloid fibrils may be made possible due to computational simulation techniques such as molecular dynamics (MD) simulation [33]–[35] and coarse-grained (CG) simulation [36]–[38], which enable the detailed insight into the mechanical deformation behavior of protein materials. For example, CG models such as elastic network model (ENM) [39]–[43] have recently been utilized to characterize the mechanical properties of supramolecular structures such as cytoskeleton crosslinker such as α-actinin rod domain [44], microtubule [45], Aβ_1–40_ amyloid fibril [46], hIAPP amyloid fibril [32], prion amyloid fibril [13], and protein crystal [47]. However, CG model may be inappropriate in order to depict sequence-structure-property relationships of protein materials due to the inability of CG model to capture the atomistic details of the mechanical deformation of protein materials. On the other hand, MD simulations are able to probe the mechanical behaviors of protein materials at atomic scales and enable the fundamental understanding of a relationship between the mechanical properties of protein materials and their molecular architecture as well as amino acid sequence [48], [49]. For instance, researchers have successfully revealed structure-property relationships of various protein materials such as titin domain [24], β sheet crystal [26], spider silk [21], collagen fibril [50], and so forth by using MD simulations.

In this work, we study the mechanical properties of hIAPP amyloid fibrils with respect to their amino acid sequence and their molecular architecture (i.e. steric zipper patterns) by using all-atom explicit water MD simulation. We consider the eight possible steric zipper patterns of hIAPP fibrils. Here, the nomenclature of steric zipper patterns is provided in a previous study by Eisenberg and coworkers [28] (see also Table 1). Our consideration of different steric zipper patterns is due to recent finding [51], [52] of two different molecular structures of hIAPP_20–29_ fibrils, which are formed based on antiparallel or parallel stacking of β strands. We have found the dependence of the mechanical properties of hIAPP fibrils on their steric zipper patterns. Moreover, we take into account the effect of genetic mutation on not only the equilibrium conformations of amyloid fibrils but also their mechanical properties. In a recent study [53], it is found that rat islet amyloid polypeptide (rIAPP) fibril does not induce the expression of type II diabetes despite the similarity between amino acid sequences of hIAPP and rIAPP except a single amino acid difference. The details of amino acid sequence for both hIAPP and rIAPP are described in Supporting Information. It has not been fully understood how single amino acid sequence difference between hIAPP and rIAPP plays a role in the mechanical properties of IAPP fibrils related to their functional role in the expression of type II diabetes. A recent study by Middleton, *et al.* [54] reports that genetic mutation critically affects the molecular structure of amyloid fibril, which highlights sequence-structure relationship of amyloid fibrils. However, to our best knowledge, sequence-structure-property relationship of amyloid fibrils has not been fully understood. We believe that our study may provide the milestone for understanding the design principles of amyloid fibrils by revealing their sequence-structure-property relationship.

**Table 1:** Classifications of polymorphic structures for hIAPP amyloid fibrils.

Class	Abbreviation	Ladder alignment	Hydrogen bond type in ladder	Zipper type
1	co-pho	co-aligned	parallel	homo
2	co-phe	co-aligned	parallel	hetero
3	aa-pho	anti-aligned	parallel	homo
4	aa-phe	anti-aligned	parallel	hetero
5	co-apho	co-aligned	anti-parallel	homo
6	co-aphe	co-aligned	anti-parallel	hetero
7	aa-apho	anti-aligned	anti-parallel	homo
8	aa-aphe	anti-aligned	anti-parallel	hetero

## Methods

### Equilibrium Dynamics Simulation

We carried out all-atom explicit water MD simulation in order to simulate the thermal fluctuation behavior of hIAPP fibril, whose structure is constructed based on the building block reported in protein data bank (pdb) with pdb code of 2KIB (for details, see Methods S1), by using NAMD package [55] along with CHARMM27 force field [56]. In particular, the equilibrium dynamics simulation of hIAPP fibril was conducted under explicit solvent condition using TIP3P water box. For the simulation of fluctuating fibril, we have performed energy minimization based on conjugate gradient method and, consequently, conducted equilibrium dynamics simulation based on NVT ensemble (for details, see Methods S1). The molecular structure of amyloid fibril was visualized by VMD software.

### Analysis of Intermolecular Interactions in Amyloid Fibril

In order to understand the thermodynamic stability of amyloid fibril, we analyze the intermolecular interaction energies in amyloid fibrils based on molecular mechanics–Poisson-Boltzmann surface area (MM-PBSA) free energy calculations [57]. Specifically, molecular mechanics energy was computed using NAMD package (energy plugin) in VMD package. The nonpolar solvation energy (Δ*G_np_*) is given by
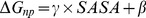
(1)where *SASA* is solvent-accessible surface area that was calculated from VMD package with using a water probe of 1.4 Å, *γ*, and *β* are parameters given as *γ* = 0.00542 kcal/mol⋅ Å^2^ and *β* = 0.92 kcl/mol. The electrostatic solvation energy (Δ*G_PB_*) was estimated by solving the linear Poisson-Boltzmann equation using Delphi v.4 software, which accounts for electrostatic interaction between solute and polarizable solvent. In PBSA calculation, partial charges were modeled using OPLSAA force field. In this work, MM-PBSA calculations were based on MD trajectories that were saved every 125 ps from the last 5 ns period in MD simulation in order to exclude unequilibrated samplings.

### Principal Component Analysis

To characterize the vibrational behavior of amyloid fibril, we employ the principal component analysis (PCA) that allows for measurement of the natural frequency of fluctuating protein structure [58]–[60]. The fluctuation matrix **Q** of amyloid fibril is defined as
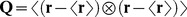
(1)where **r** is the coordinates of Cα atoms constituting an amyloid fibril, an angle bracket indicates the ensemble average, and a symbol 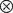 represents tensor product. Note that the fluctuation matrix **Q** is the 3*N* ×3*N* matrix, where *N* is the total number of Cα atoms. The stiffness matrix **K** of amyloid fibril can be obtained from statistical mechanics theory such as



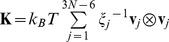
(2)Here, *ξ_j_* and **v** *_j_* represent the *j*-th eigenvalue and its corresponding normal mode vector, respectively, of fluctuation matrix **Q**, *k_B_* is the Boltzmann’s constant, and *T* is the absolute temperature. Here, the eigenvalues and their corresponding normal modes of fluctuation matrix **Q** were obtained using spectral decomposition based on component mode synthesis as described in our previous work [61]. Based on quasi-harmonic analysis, the natural frequency of amyloid fibril can be computed as follows.
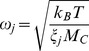
(3)where *ω_j_* is the natural frequency of amyloid fibril at the *j*-th mode, and *M_C_* is the molecular weight of Cα atom. The details of the method are presented in Supporting Information. Here, PCA calculations were based on MD trajectories that were recorded at every 6 ps from the last 5 ns period in MD simulation in order to include only equilibrated samples.

### Analysis on Thermal Fluctuation of Amyloid Fibril

To understand the thermal fluctuation behavior of an amyloid fibril, we consider the root-mean-square fluctuation (RMSF), bending angle, and dihedral angle, which are described in Methods S1.

In order to quantitatively depict the contribution of *k*-th normal mode to the thermal fluctuation of a fibril, we introduce a dimensionless parameter *α_k_*, which is defined as
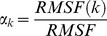
(4)where *RMSF* is a root-mean-square fluctuation for vibrating amyloid fibril (see Methods S1), and *RMSF*(*k*) represents the contribution of *k*-th normal mode to the root-mean-square fluctuation. Here, *RMSF* is given by



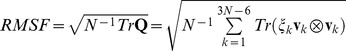
(5)The contribution of *k*-th normal mode to the root-mean-square fluctuation is given as
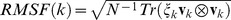
(6)


As a consequence, the dimensionless parameter *α_k_* is represented in the form
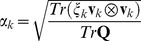
(7)


For validating the thermodynamic stability of amyloid fibril, we take into account order parameter (OP) defined as [30]

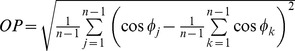
(8)where *n* is the total number of β sheet layers constituting an amyloid fibril, and *φ_j_* is the dihedral angle of the *j*-th β sheet layer. The structural stability of amyloid fibril is checked based on OP such that a fibril structure with its OP larger than 0.07 is regarded as an unstable and disordered structure [30].

### Continuum Elastic Model

In order to relate the natural frequencies of amyloid fibril computed from atomistic simulations to the mechanical properties (e.g. bending rigidity, axial elastic modulus, and torsional shear modulus) of amyloid fibril, we consider a continuum elastic beam model, particularly the Euler-Bernoulli beam model [62], which is useful in describing the mechanical behavior of one-dimensional nanostructures [63]. The equation of motion for vibrating amyloid fibril modeled as an Euler-Bernoulli beam model is represented in the form of
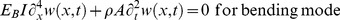
(4.a)




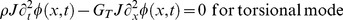
(4.b)




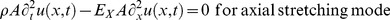
(4.c)where *E_B_*, *I*, *ρ*, *A*, *J*, *G_T_*, and *E_X_* represent the bending elastic modulus, cross-sectional moment of inertia, mass density, cross-sectional area, cross-sectional polar moment of inertia, torsional shear modulus, and axial elastic modulus, respectively, of an amyloid fibril, *w*(*x*, *t*), *φ*(*x*, *t*), and *u*(*x*, *t*) indicate the transverse deflection, twist angle, and axial displacement, respectively, as a function of coordinate *x* (along the longitudinal direction of fibril) and time *t*. Note that the persistent length (*l_p_*) of amyloid fibril is given as *l_p_* = *E_B_I*/*k_B_T*, where *k_B_* and *T* are Boltzmann’s constant and absolute temperature, respectively. Based on Eq. (4), the natural frequencies of amyloid fibril are given by



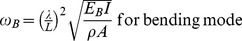
(5.a)

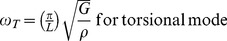
(5.b)




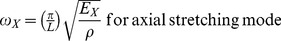
(5.c)Eq. (5) clearly demonstrates that the elastic moduli of amyloid fibril can be obtained from its natural frequencies that can be computed from all-atom explicit water MD simulation.

## Results

### Equilibrium Conformation of Amyloid Fibril

**Figure 1:**
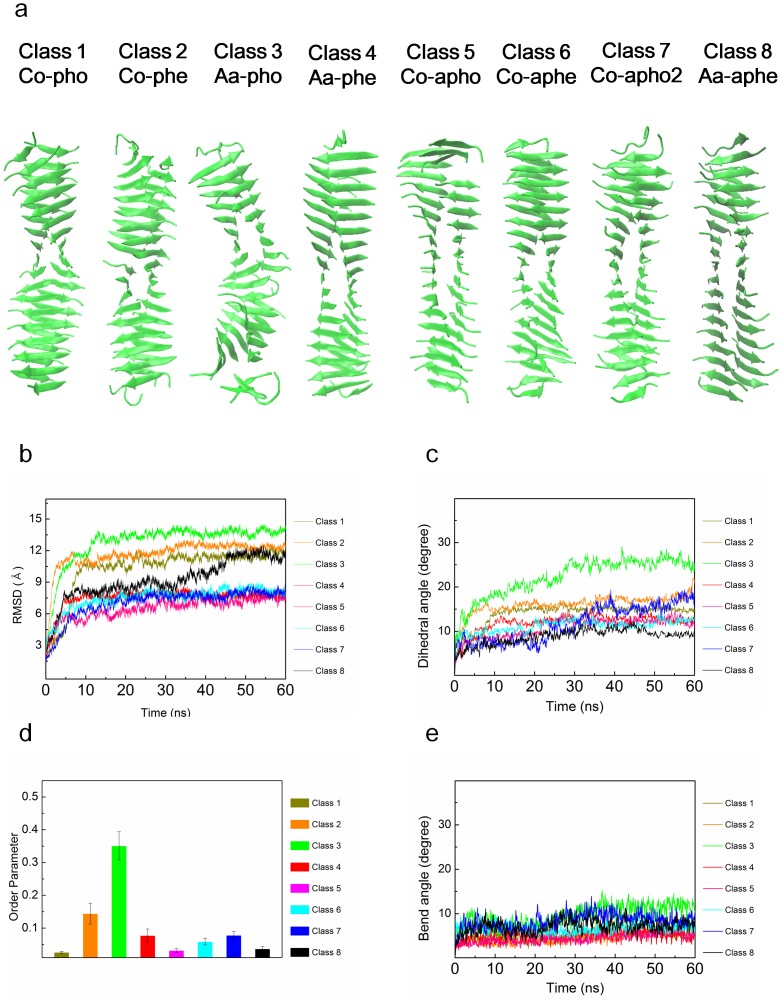
Equilibrium dynamics of wild type hIAPP fibrils. (**a**) Conformations of polymorphic hIAPP fibrils at time of 60 ns obtained from explicit water MD simulation. (**b**) Root-mean-square distance (RMSD) for polymorphic hIAPP fibrils as a function of time. (**c**) Dihedral angles of hIAPP fibrils with respect to time. (**d**) Order parameters of polymorphic hIAPP fibrils. (**e**) Bending angles of hIAPP fibrils as a function of time.

In order to gain insight into the energetically favorable configurations of hIAPP amyloid fibrils among their eight possible steric zipper patterns, we consider the thermodynamic stability of hIAPP fibrils with their eight possible polymorphic structures. To check the thermodynamic stability, we performed equilibrium dynamics simulations of hIAPP fibrils (with their 8 polymorphic structures) for a period of 60 ns. Here, it should be noted that MD simulation with timescale of 60 ns may be sufficient to obtain the equilibrated molecular structures of amyloid fibrils. In previous studies [30], [31], [64], the simulation timescale of 20 ns was used to acquire the equilibrated molecular structures of amyloid fibrils. **Figure 1a** shows the conformations of polymorphic hIAPP fibrils at the time of 60 ns. As shown in **Figure 1a**, all polymorphic structures for hIAPP fibril seem to be thermodynamically stable. In order to quantitatively characterize the stability of such polymorphic structures, we take into account the RMSDs of polymorphic hIAPP fibrils. **Figure 1b** provides that after the time of ∼10 ns, the most of RMSDs reach a steady-state value, which indicates that polymorphic structures for hIAPP fibril may be thermodynamically stable after 10 ns. Remarkably, it is shown that the steady-state value of RMSD for anti-aligned parallel homo (aa-pho) structure is different from that of other polymorphic structures. This may imply that this structure exhibits the different equilibrium conformation from that of other polymorphic structures.

Moreover, we consider a twist angle (dihedral angel) between β sheet layers constituting a hIAPP fibril as a function of time (**Figure 1c**). It is found that after the time of ∼5 ns, the dihedral angles of polymorphic structures except aa-pho and co-apho2 structures approach to steady-state values, indicating that these polymorphic structures are energetically favorable. For aa-pho and co-apho2 structures, the dihedral angles reach steady-state values after time of 30 ns. This is consistent with our previous finding based on RMSDs as described above. It is interestingly found that aa-pho structure exhibits the equilibrium dihedral angle of ∼25°, whereas the equilibrium dihedral angle of other polymorphic structures is given by ≤15°. This clearly suggests that hIAPP fibrils can possess the two different equilibrium conformations based on steric zipper patterns. In particular, the equilibrium dihedral angle (i.e. ∼10°) of co-aphe, co-apho, and aa-aphe structures suggests that 72 β strands form a helical pitch of these fibril structures, which is consistent with the molecular structure of hIAPP fibril observed by solid-state NMR [51]. On the other hand, based on equilibrium dihedral angle, the helical pitch of aa-pho structure is formed by 28 β strands. Our finding suggests that the equilibrium structures of hIAPP fibrils are determined by their steric zipper patterns, that is, chemical interaction pattern between β sheet layers. In other words, the thermodynamically favorable structure of amyloid fibril is inherently encoded in the steric zipper patterns. Moreover, we investigate the OPs of all polymorphic structures in order to check the thermodynamic stability of such polymorphic structures. As shown in **Figure 1d**, it is found that the OPs of all polymorphic structures are estimated as ≤0.04, which suggests that all polymorphic hIAPP fibrils are thermodynamically stable. In addition, we scrutinize the bending angle of all polymorphic structures for hIAPP fibrils as a function of time, since the thermal fluctuation of a one-dimensional biological fiber such as amyloid fibrils is significantly attributed to the bending motion (see below). **Figure 1e** depicts that after ∼10 ns, the bending angles of all polymorphic amyloid fibrils fluctuate around the equilibrium value of ≤10° depending on the polymorphic structures.

For gaining insight into the thermodynamic stability of amyloid polymorphic structures, we take into account the MM-PBSA calculations. As shown in Table 2, the electrostatic solvation energy (Δ*G_PB_*) mostly contributes to solvation free energy in amyloid fibril. It is shown that the free energies of amyloid fibrils formed based on parallel stacking are higher than those of fibrils constructed based on antiparallel stacking, which suggests that anti-parallel stacking to form a fibril is thermodynamically favorable. This is consistent with finding that OPs of fibrils made based on parallel stacking are larger than those of fibrils formed based on antiparallel stacking. In particular, aa-pho structure exhibits the highest solvation free energy (−47273.6 kcal/mol) and highest OP (0.035) among all polymorphic structures. This suggests that the fibril formed based on anti-aligned parallel homo (aa-pho) structure is thermodynamically unstable when compared with other fibril structures.

**Table 2:** MM-PBSA free energy calculations.

Polymorphic structure	Molecular mechanics (MM) energy (kcal/mol)	Nonpolar solvation energy (kcal/mol)	Electrostatic solvation energy (kcal/mol)	Total energy (kcal/mol)
co-pho	−3062.66±619.54	−182.84±33.89	−45230.2±63.39	−48475.7
co-phe	−1709.18±684.00	−184.47±35.06	−45860.3±34.43	−47754.0
aa-pho	−970.42±684.11	−181.71±34.54	−46121.5±60.56	−47273.6
aa-phe	−2100.02±662.25	−186.57±35.28	−45150.3±5.17	−47436.9
co-apho	−5021.42±655.05	−188.28±34.35	−44564.3±66.37	−49774.0
co-aphe	−5338.77±687.69	−185.47±35.11	−44913.9±48.48	−50438.1
aa-apho	−5376.54±682.24	−185.65±35.44	−44290.4±20.62	−49852.6
aa-aphe	−5508.94±770.62	−180.718±35.83	−45048.5±16.79	−50738.2

### Vibrational Characteristics of Polymorphic hIAPP Fibrils

As elucidated in Section 2.1, the estimation of the vibrational characteristics of a material is a useful route to characterizing the mechanical properties (particularly, elastic moduli) of the material. For instance, in a recent study [65], the mechanical properties (e.g. persistent length) of microtubule have been measured using spectral decomposition method along with continuum elastic model. A previous study by Wang, *et al.* [66] has also utilized the spectral decomposition theory with wormlike chain (WLC) model in order to characterize the mechanical properties of hemoglobin fiber. This spectral decomposition theory with WLC model has recently been extended for studying the mechanical properties (e.g. persistent length) of amyloid fibrils based on their structures acquired from cryo-electron microscopy [67]. Moreover, a recent study by Zewail and coworkers [9] reports the elastic moduli of Aβ amyloid fibrils based on measurement of their vibrational properties using 4D electron microscopy. These previous studies [9], [65]–[67] suggest that characterization of the vibrational properties of biological materials allows for extracting their mechanical properties.

In order to verify the ability of continuum elastic model (i.e. Euler-Bernoulli beam model) to dictate the vibrational behavior of amyloid fibrils, we consider the deformation modes of hIAPP fibrils obtained from all-atom explicit water MD simulation. It is shown that the vibrational modes of hIAPP fibrils are able to depict bending modes, stretching mode, and torsional mode, respectively (see **Figure S1**). Here, we found two bending modes for hIAPP fibril, which are attributed to anisotropic cross-sectional moments of inertia for hIAPP fibril. In particular, there are two principal cross-sectional moments of inertia for hIAPP fibril. These two bending modes are well fitted to the mode shape of Euler-Bernoulli beam model given as *φ_n_*(*x*) = *A_n_*[(cosh*β_n_x*+cos*β_n_x*) – *σ_n_*(sinh*β_n_x*+sin*β_n_x*)] (for details, see refs. [32], [68]), which suggests that the vibrational characteristics of hIAPP fibril obtained from all-atom explicit water MD simulation are well dictated by a continuum elastic beam model.

**Figure 2:**
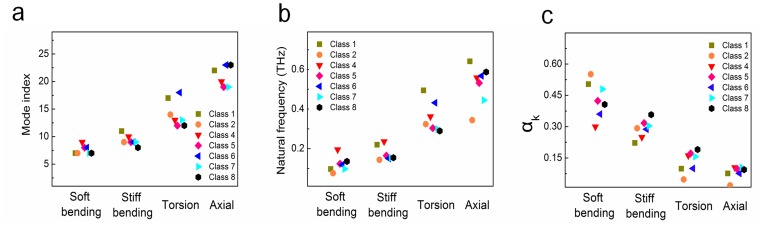
Vibrational characteristics of polymorphic hIAPP fibrils. (**a**) Mode indices for the deformation modes of hIAPP fibrils. (**b**) Natural frequencies of polymorphic hIAPP fibrils at a given deformation mode. (**c**) Contribution of each deformation mode to the thermal fluctuation of polymorphic hIAPP fibrils.

**Figure 2a** shows the mode indices of all polymorphic hIAPP fibrils for their specific deformation modes such as bending, stretching, and torsional modes. Here, it should be noted that first six zero modes correspond to the rigid body motions of hIAPP fibrils. It is found that two bending deformation modes for all polymorphic hIAPP fibrils correspond to low-frequency normal modes (i.e. 7^th^∼10^th^ normal mode) regardless of the steric zipper patterns of hIAPP fibrils. On the other hand, other deformation modes such as stretching and torsional modes for hIAPP fibrils are high-frequency normal modes. This indicates that the thermal fluctuation behavior of polymorphic hIAPP fibrils is attributed to their bending motion, since low-frequency motion determines the thermal fluctuation behavior of proteins (for more details, see below and **Figure 2c**). This is consistent with theoretical model, e.g. WLC model [69], which delineates that the deformation behavior of biological fibers such as DNA [70]–[72], protein [73], [74], and microtubule [16] can be described by their bending motion rather than any other deformation modes such as stretching and torsion. Moreover, we found that the high-frequency deformation modes (i.e. stretching and torsional modes) are dependent on the steric zipper patterns of hIAPP fibrils. This indicates that the torsional and stretching deformations of amyloid fibril are attributed to its high-frequency mode that depends on the steric zipper pattern, whereas the bending motion of amyloid fibril is due to its low-frequency mode independent of chemical interaction patterns between β sheet layers. **Figure 2b** provides the natural frequencies of hIAPP fibrils corresponding to their deformation modes such as bending, stretching, and torsional modes, respectively. It is shown that the natural frequencies of hIAPP fibrils for their soft bending mode are estimated in a range of 0.1 THz to 0.2 THz, and that the natural frequencies for soft bending mode are highly dependent on the steric zipper pattern of hIAPP fibrils. It is shown that hIAPP fibrils formed by antiparallel stacking of β strands exhibit the natural frequency of ∼0.2 THz, which is larger than that (i.e. ∼0.1 THz) of fibrils formed by parallel stacking of β strands, for soft bending mode. This implies that antiparallel stacking of β strands enhances the bending rigidity of amyloid fibril, since the natural frequency is linearly proportional to the square root of elastic modulus. This provides that the steric zipper pattern is a key design parameter that determines the mechanical properties of amyloid fibrils.

In order to gain more insight into a relationship between the deformation modes and the thermal fluctuation behavior of hIAPP fibrils, we introduce the dimensionless parameter *α_k_*, which measures the contribution of *k*-th normal mode to the thermal fluctuation of amyloid fibril. As shown in **Figure 2c**, it is found that the contribution of soft bending mode to the thermal fluctuation of hIAPP fibrils is estimated in a range of 20% to 60% depending on their steric zipper patterns. This clearly elucidates the role of the steric zipper pattern on the thermal fluctuation behavior of amyloid fibrils. In a similar manner, the contribution of stiff bending mode to the thermal fluctuation of hIAPP fibrils is measured in a range between 15% and 45% depending on the steric zipper pattern. It is found that two bending modes contribute to more than 60% of thermal fluctuation for hIAPP fibrils, which is consistent with previous findings [41], [75], [76] that low-frequency motion determines the thermal fluctuation behavior of protein molecule. On the other hand, it is remarkably shown that the contribution of torsional mode and stretching mode to the thermal fluctuation of hIAPP fibrils is estimated as ∼15% and ∼10%, respectively, regardless of the steric zipper pattern of hIAPP fibril. This suggests that the contribution of high-frequency motion to the thermal fluctuation behavior of amyloid fibrils is almost independent of their steric zipper pattern, which is a key design parameter that determines the vibrational (mechanical) behavior of amyloid fibrils.

### Polymorphism-Dependent Mechanical Properties

**Figure 3:**
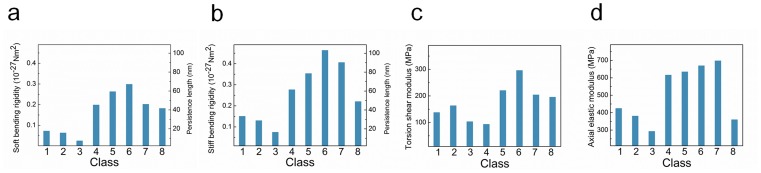
Mechanical properties of polymorphic hIAPP fibrils. (**a**) Soft bending rigidity, (**b**) stiff bending rigidity, (**c**) torsional shear modulus, and (**d**) axial elastic modulus for polymorphic hIAPP fibrils.

Based on continuum mechanics theory that relates the natural frequency of amyloid fibrils obtained from all-atom explicit water MD simulation to their mechanical properties, we measure the mechanical properties (i.e. bending rigidity, axial elastic modulus, and torsional shear modulus) of hIAPP fibrils as a function of their steric zipper patterns (**Figure 3**). The elastic moduli of amyloid fibrils corresponding to their bending modes are estimated in a range of ∼1 GPa to ∼10 GPa depending on the steric zipper patterns of hIAPP fibrils. These bending elastic moduli are comparable to those of Aβ_1–40_ amyloid fibrils measured by AFM imaging-based experiment [14] and normal mode analysis (NMA) based on coarse-grained model [46]. Here, it should be noted that the bending elastic modulus of hIAPP fibril, whose length is ∼12 nm in this work, is much less than that of hIAPP fibrils, whose length is >50 nm considered in our previous work [32]. This is ascribed to the shear effect that plays a critical role on the bending deformation of short amyloid fibrils. The torsional shear moduli of hIAPP fibrils are measured in a range of ∼0.1 to ∼0.3 GPa, which is smaller than the shear modulus of Aβ_1–40_ amyloid fibril computed from coarse-grained model [46]. The axial elastic moduli of hIAPP fibrils are evaluated as ∼0.4 to ∼0.7 GPa, which is one order of magnitude smaller than the elastic modulus of Aβ_1–40_ amyloid fibril measured from MD simulation [77]. More remarkably, as shown in **Figure 3**, the steric zipper pattern is a key design parameter that controls the mechanical properties of amyloid fibrils. Specifically, the bending elastic moduli of hIAPP fibrils are highly dependent on their steric zipper pattern such that the soft bending rigidities of hIAPP fibrils formed by parallel stacking of β strands are measured as ∼10^–28^ N⋅m^2^, while hIAPP fibrils made based on antiparallel stacking of β strands exhibit soft bending rigidity of ∼2×10^–28^ N⋅m^2^ (**Figure 3a**). This indicates that the optimal bending rigidity of amyloid fibrils can be achieved through antiparallel stacking of β strands. This finding is consistent with previous studies [26], [32] reporting that antiparallel stacking of β strands to construct β sheet-rich protein materials enhances their bending rigidity, which is attributed to the effect of geometric confinement of chemical bonds (e.g. hydrogen bonds) between β strands on the mechanical properties of β sheet-rich protein materials. However, the torsional shear moduli of hIAPP fibrils are not significantly dependent on the steric zipper patterns. This suggests that antiparallel stacking of β strands is only effective to increase the bending rigidity of amyloid fibrils but is ineffective to maximize their torsional rigidity. This implies that structure-property relationships of amyloid fibrils are governed by the deformation mode (i.e. loading mode).

In order to fully understand the role of the steric zipper pattern on the mechanical properties of hIAPP fibrils, we measure the hydrogen (H) bond per donor (residue) for all polymorphic structures as a function of time (**Figure S2**). Our consideration of H-bond per residue is attributed to recent finding [24], [25], [78] that the mechanical strength of a protein molecule is determined from H-bonds and their configuration. It is shown that the magnitude of fluctuation for H-bond per residue is much smaller than the ensemble average of H-bond per donor, which implies that during the thermal fluctuation of polymorphic hIAPP fibrils, the significant rupture or formation of H-bonds is not likely to occur; in general, the rupture of H-bonds critically affects the mechanical response (e.g. stiffness) of a protein molecule [24]. It is shown that the ensemble average of H-bond per residue for co-apho2 structure is measured as ∼0.4, which is larger than that (i.e. ∼0.25) of co-pho structure. This suggests that antiparallel stacking allows amyloid fibril to exhibit the optimal density of H-bonds between β strands. Moreover, based on the H-bond networks of all polymorphic structures (i.e. 8 steric zipper patterns) as shown in **Figure S4**, it is found that the directions of all H-bonds for polymorphic amyloid fibrils are parallel to the fibril axis, which suggests that the dependence of the mechanical properties of amyloid fibrils on their polymorphic structures is attributed to the density of H-bonds rather than the configuration of H-bond network. In addition, we have also shown that the H-bonds of amyloid fibril are likely to be conserved during the thermal fluctuation (**Figure S6**). This suggests that the steric zipper patterns determines the density of H-bonds between β strands, and consequently, the mechanical behavior of amyloid fibrils.

### Effect of Genetic Mutation on the Equilibrium Conformation of hIAPP Fibrils

We study the effect of single point mutation of hIAPP_20–29_ constituting a hIAPP fibril on its molecular structure, since rat islet amyloid polypeptide (rIAPP) fibril does not induce any type II diabetes in a rat despite similarity between amino acid sequences of hIAPP_20–29_ and rIAPP [53]. In particular, the sequence of hIAPP_20–29_ is given as “SNNFGAILS”, while the amino acid sequence of rIAPP is suggested as “SNNLGAILS”. In a recent decade, a rIAPP chain was regarded as an inhibitor [53], which inhibits the formation of hIAPP fibrils. However, a recent study [54] reports that rIAPP can be also reacted with hIAPP chains to form an amyloid fibril, while the reaction rate between rIAPP and hIAPP chains is slower than that between hIAPP chains. In other words, rIAPP chain is also able to form an amyloid fibril but the aggregation process to form a fibril is very slow. Moreover, it is shown that an amyloid fibril synthesized with rIAPP chains exhibits a different molecular structure from that of fibril made of hIAPP chains [54]. This observation has led us to study the equilibrium conformations of both hIAPP and rIAPP fibrils, which can provide an insight into the effect of genetic mutation on the molecular structure of amyloid fibril.

**Figure 4:**
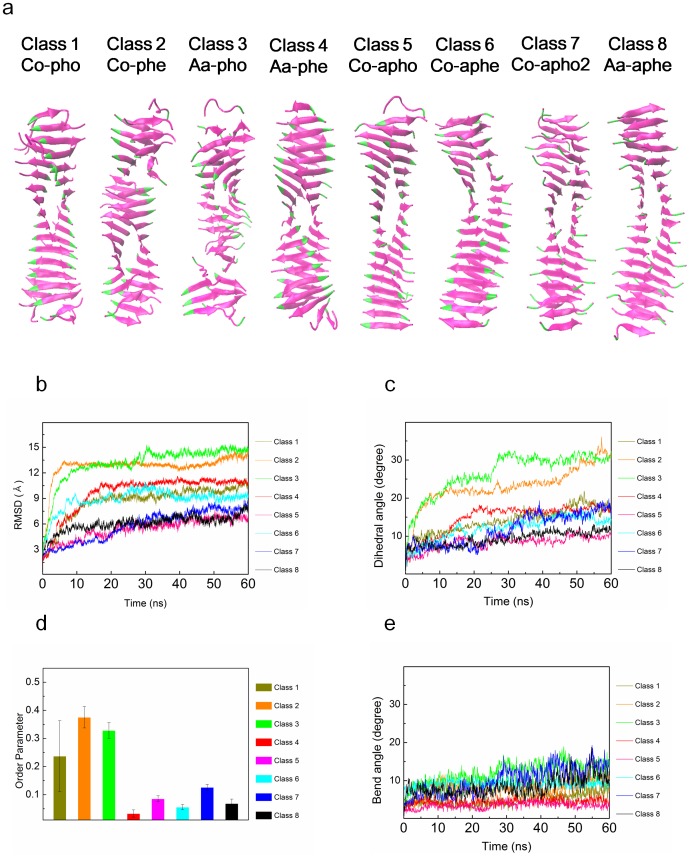
Effect of a single point mutation on the equilibrium dynamics of polymorphic hIAPP fibrils. (**a**) Conformations of mutated hIAPP fibrils at time of 60 ns obtained from explicit water MD simulation. (**b**) Root-mean-square distance (RMSD) for mutated fibrils with respect to time. (**c**) Dihedral angles of mutated hIAPP fibrils as a function of time. (**d**) Order parameters for mutated fibrils with respect to their polymorphism. (**e**) Bending angles for mutated fibrils as a function of time.

**Figure 4a** illustrates the conformations of polymorphic rIAPP fibrils that were obtained from all-atom explicit water MD simulation at time of 60 ns. It is shown that co-phe and aa-pho structures for mutated fibril might be thermodynamically unstable, since the rIAPP chains at the end of these fibrils are severely distorted (**Figure 4a**), which may be a signature of unstable structure. It is also found that these two polymorphic structures exhibit the distorted neutral axis, indicating that these two polymorphic structures are not straightly formed along the fibril axis. This also suggests that these two polymorphic structures are thermodynamically unfavorable. **Figure 4b** depicts the RMSDs of polymorphic rIAPP fibrils as a function of time. It is found that for co-phe and aa-pho structures, the mutation increases RMSDs by the amount of ∼2 Å, even though RMSDs approach to a steady-state value after 30 ns. This suggests that RMSD alone is not sufficient to verify the thermodynamic stability of amyloid fibrils. As shown in **Figure 4c**, it is remarkably found that the dihedral angles of these two polymorphic structures are increasing with time even up to 30°, which indicates the instability of these polymorphic structures. Here, it should be noted that the maximum dihedral angle of wild type fibril is less than 30°. It is shown that the equilibrium dihedral angle of these polymorphic mutated fibrils (except co-phe and aa-pho structures) is slightly different from ∼10° that is the equilibrium dihedral angle of wild type (WT) fibrils (i.e. hIAPP fibrils). This implies that a single point mutation of hIAPP_20–29_ affects the equilibrium conformations of amyloid fibrils, which sheds light on sequence-structure relationship of amyloid fibrils. Furthermore, we investigate the OPs of polymorphic mutated fibrils. It is shown in **Figure 4d** that for co-phe structure, the single point mutation significantly increases OP from ∼0.15 to ∼0.6. For aa-pho structure, the mutation increases OP from ∼0.35 to ∼0.4, which is much larger than ∼0.07, an indicative of thermodynamic instability [30]. In addition, the genetic mutation increases the OP of co-pho structures by the amount of ∼0.2. This suggests that the genetic mutations increases the instability of co-pho, co-phe, and aa-pho fibril structures.

### Effect of Genetic Mutation on the Mechanical Properties of Polymorphic Fibrils

**Figure 5:**
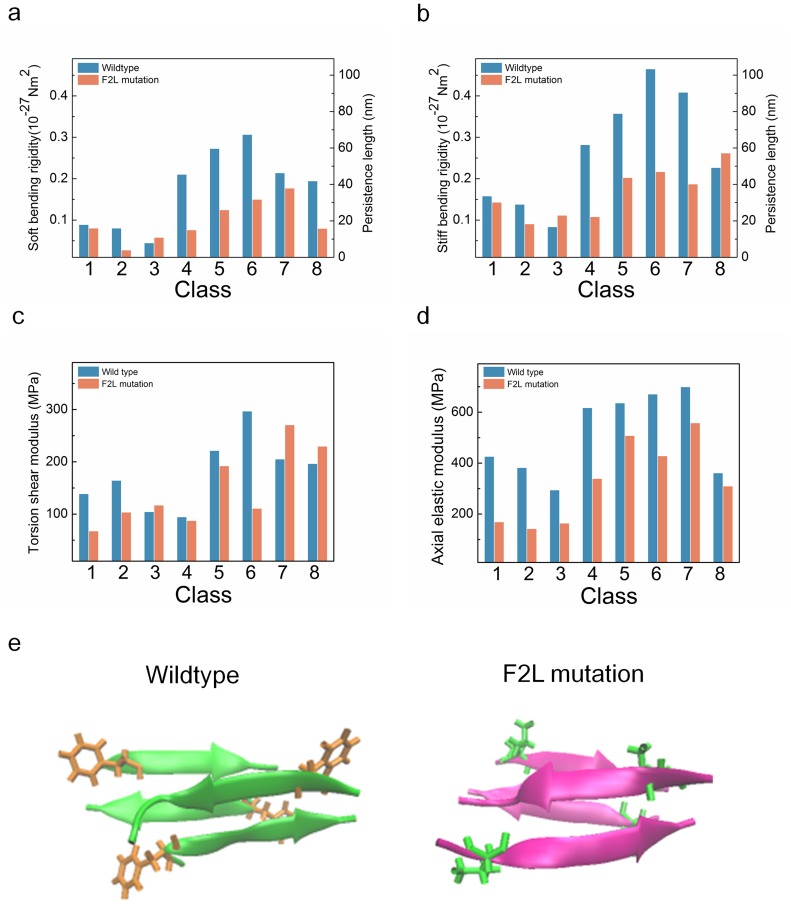
Mechanical properties of mutated hIAPP fibrils: **(a)** soft bending rigidity, **(b)** stiff bending rigidity, **(c)** torsional shear modulus, and **(d)** axial elastic modulus. (**e**) Intermolecular interactions between β sheet layers for wild type hIAPP fibril and mutated fibril of class 5. The hydrogen bond networks colored in red, and the side chain is shown with colored in yellow (hydrogen), red (oxygen), blue (nitrogen) and cyan (carbon).

Since the genetic mutation critically affects the equilibrium structures of IAPP fibrils, we study the mechanical properties of mutated fibrils for gaining insight into the role of genetic mutation on the mechanical properties of amyloid fibrils. **Figure 5a** shows the bending rigidities of both WT and mutated fibrils with respect to their steric zipper patterns. It is shown that a single point mutation does not significantly affect the soft bending rigidities of amyloid fibrils formed by parallel stacking of β strands, whereas the soft bending rigidities of co-apho, co-aphe, and aa-aphe fibril structures formed by antiparallel stacking of β strands are critically reduced by a single point mutation. This suggests that the alteration of the bending rigidity of amyloid fibrils due to genetic mutation is dependent on their steric zipper patterns. In other words, the mutation-driven change of amyloid mechanics may be inherently encoded in the molecular architecture (i.e. steric zipper pattern) of amyloid fibril. In addition, the F2L mutation critically decreases the stiff bending rigidities of co-apho, co-aphe, and co-apho2 fibril structures. Except aa-pho and aa-aphe structures, the genetic mutation significantly decreases the stiff bending rigidities of fibrils. Here, for aa-pho and aa-aphe structures, the mutation slightly increases the stiff bending rigidities of fibrils. This suggests that genetic mutation mostly degrades the bending rigidity of amyloid fibril. It is found that the genetic mutation decreases the torsional shear moduli of fibrils except aa-pho, aa-phe, co-apho2, and aa-aphe structures (**Figure 5c**). It is shown in **Figure 5d** that the genetic mutation reduces the axial elastic moduli of IAPP fibrils regardless of their steric zipper patterns. This suggests that the genetic mutation is a useful route to degrading the axial elastic properties of amyloid fibrils. In summary, the genetic mutation affects the mechanical properties of amyloid fibrils depending on their steric zipper patterns. Specifically, the alteration of bending and torsional elastic properties of amyloid fibrils due to genetic mutation depends on their steric zipper patterns, while the genetic mutation reduces the axial elastic moduli of amyloid fibrils regardless of their steric zipper patterns.

In order to understand the effect of mutation on the mechanical properties of amyloid fibrils, we investigate chemical interaction between β sheet layers constituting an amyloid fibril. First, we scrutinized the role of genetic mutation on H-bond per residue as a function of time, since H-bond interaction between β sheet layers is a key design parameter that determines the mechanical behavior of a protein molecule as described above. It is found that the genetic mutation does not significantly alter the H-bond per residue for polymorphic hIAPP fibrils regardless of their steric zipper patterns (**Figure S3**). In particular, as shown in **Figure S5**, the F2L mutation does not critically change the number of H-bonds and H-bond network. Moreover, during the thermal fluctuation, the H-bond network is conserved (**Figure S7)**. This suggests that H-bond is not a key factor that governs the mutation-driven change of the mechanical behavior of amyloid fibrils. On the other hand, as shown in **Figure 5e**, the genetic mutation results in significant alteration of aromatic interactions between β sheet layers constituting a hIAPP fibril. In particular, the aromatic rings of a side chain for β sheet layer are present in WT amyloid fibrils, while the genetic mutation eliminates aromatic interactions between β sheet layers. This indicates that aromatic interaction depending on the amino acid sequence of fibril is a key factor that determines the mechanical properties of hIAPP fibrils. In other words, sequence-dependent side chain interaction plays a role in both the equilibrium conformations of polymorphic hIAPP fibrils and their mechanical properties. Our finding is qualitatively consistent with recent studies [79]–[81] reporting that the equilibrium conformations of hIAPP fibrils are significantly dependent on aromatic interactions between β sheet layers. In summary, the F2L mutation reduces the mechanical properties of hIAPP amyloid fibril, which is attributed to the mutation-driven elimination of aromatic interaction. It should be noted that the genetic mutation does not always decrease the mechanical properties of amyloid fibrils. For instance, genetic mutation leading to formation of salt bridge can result in increasing the elastic moduli of amyloid fibrils [82]. Specifically, an Aβ amyloid fibril without salt bridges twists dramatically, whereas that with salt bridges is mechanically rigid. This suggests that genetic mutation is a useful route in tuning the mechanical properties of amyloid fibril. Our study highlights sequence-structure-property relationship of amyloid fibrils, which may provide key design principles showing that the properties of amyloid fibrils can be tuned based on sequence-structure-property relationships necessary for enabling the development of not only molecular therapeutics but also biomimetic materials.

## Discussion

In this work, we have studied the mechanical properties of amyloid fibrils using all-atom explicit water MD simulation along with continuum mechanics theory. Our study shows that the mechanical properties of amyloid fibrils are closely related to their molecular architectures, particularly the steric zipper patterns, and that the structure-dependent mechanical properties of amyloid fibrils are critically affected by genetic mutation. Specifically, amino acid sequence determines chemical interaction between β sheet layers constituting an amyloid fibril, and consequently, its mechanical properties. Our study sheds light on sequence-structure-property relationship of amyloid fibril, which highlights the design principles that provide an insight into how to tune the mechanical properties of an amyloid fibril based on its molecular structure and sequence.

In order to further understand the thermal fluctuation behavior of amyloid fibrils, which can be observed by AFM experiment, e.g. see refs. [14], [22], it is essential to study the mechanical (e.g. fluctuation) behavior of amyloid fibrils in the physiological condition. In our all-atom MD simulation, we excluded the effect of pH, ion, and temperature on the mechanical behavior of amyloid fibrils. It should be noted that physiological conditions (e.g. pH, ion concentration, temperature, etc.) may play a key role in not only the equilibrium conformations of amyloid fibrils but also their mechanical properties. In particular, in our recent study [83], it is found that a chemical condition (e.g. pH) of a solvent determines electrostatic interaction between amyloidogenic core and amyloid oligomer, which leads to the dependence of the equilibrium structures of amyloid fibrils on a physiological condition such as pH. Moreover, in a recent study [5], it has been reported that the metal ion is a key factor that affects an aggregation mechanism to form an Aβ amyloid fibrils as well as their neurotoxicity. In particular, the high concentration of Al^3+^ ions has been observed in patients who suffer from Alzheimer’s diseases [84], [85], even though the role of Al^3+^ ion on pathogenesis is still controversial [86]. Moreover, it has recently been provided that Cu^2+^ ions makes the polymorphic Cu^2+^ complexes with Aβ amyloids, which is attributed to the fact that there are several possible binding sites for Cu^2+^ ions in the Aβ amyloid fibrils [87]. These observations indicate the role of metal ions on the polymorphic structures of amyloid fibrils as well as their mechanical properties in a physiological condition. In summary, the effect of physiological condition on the mechanical properties of amyloid fibrils has not been thoroughly studied yet; it is necessary to understand the mechanical behavior of pathological amyloids in a physiological condition, which can provide an insight into how to effectively treat the pathological amyloids for future therapeutics by chemically changing the mechanical properties of an amyloid fibril. The role of physiological condition on the polymorphic structures of amyloid fibrils and their mechanical behaviors will be taken into account for our future study.

In conclusion, we provide sequence-structure-property relationships of amyloid fibrils, which allow us to understand how the mechanical properties of amyloid fibrils are encoded in their molecular structures and their amino acid sequences. Specifically, as suggested in our work, the bending rigidity of an amyloid fibril can be tuned by changing the molecular structure (i.e. steric zipper pattern); the remarkable bending rigidity of amyloid fibril can be achieved through antiparallel β strands that maximize the density of H-bond per residue, which is a key factor determining the mechanical properties of a protein molecule. Moreover, our simulation results show that genetic mutation critically affects the equilibrium conformations of polymorphic amyloid fibrils and their mechanical properties. This sheds light on the role of amino acid sequence on the molecular structure of amyloid fibrils and their mechanical properties. Our study unveils the design principle of amyloid fibril, which shows that chemical interaction between β strands depending on the amino acid sequence of amyloid fibril governs the mechanical properties of amyloid fibril.

## Supporting Information

Figure S1 Deformation modes of human islet amyloid polypeptide (hIAPP) fibril obtained from explicit water molecular dynamics simulations.
(TIF)

Figure S2 H-bonds per residue for polymorphic hIAPP fibrils.
(TIF)

Figure S3 H-bonds per residue for mutated IAPP fibrils with their polymorphic structures.
(TIF)

Figure S4 H-bond network for wild type fibril. Here, hydrogen bonds are indicated by red dotted lines, while hydrogen, oxygen, nitrogen, and carbon atoms are colored in yellow, red, blue, and cyan, respectively.
(TIF)

Figure S5 H-bond network for mutated fibril.
(TIF)

Figure S6 Hydrogen bond network for wild type fibrils that undergo deformation modes.
(TIF)

Figure S7 Hydrogen bond networks for mutated fibrils undergoing deformation modes.
(TIF)

Methods S1 Detailed supporting methods for the model of an amyloid fibril, equilibrium molecular dynamic simulation, and characterization of equilibrium dynamics of fluctuating amyloid fibrils.
(DOCX)
