# Supplementary figures and images for: Role of Sequence and Structural Polymorphism on the Mechanical Properties of Amyloid Fibrils
Source: PLoS One. 2014 Feb 14;9(2):e88502. doi: 10.1371/journal.pone.0088502 (PMC3925137; doi:10.1371/journal.pone.0088502)

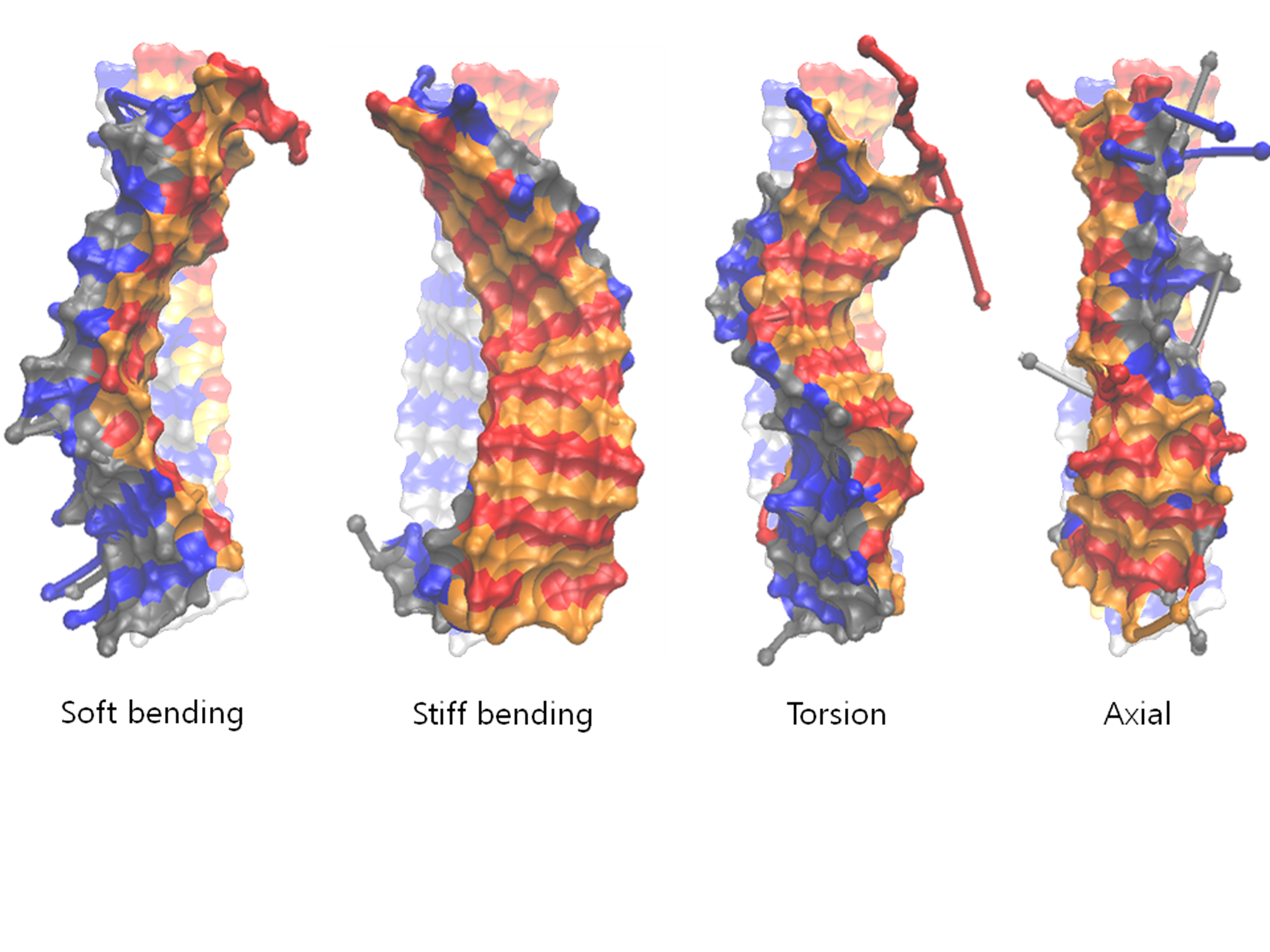

Supplement: Figure S1 — Deformation modes of human islet amyloid polypeptide (hIAPP) fibril obtained from explicit water molecular dynamics simulations. (TIF) [file pone.0088502.s001.tif]

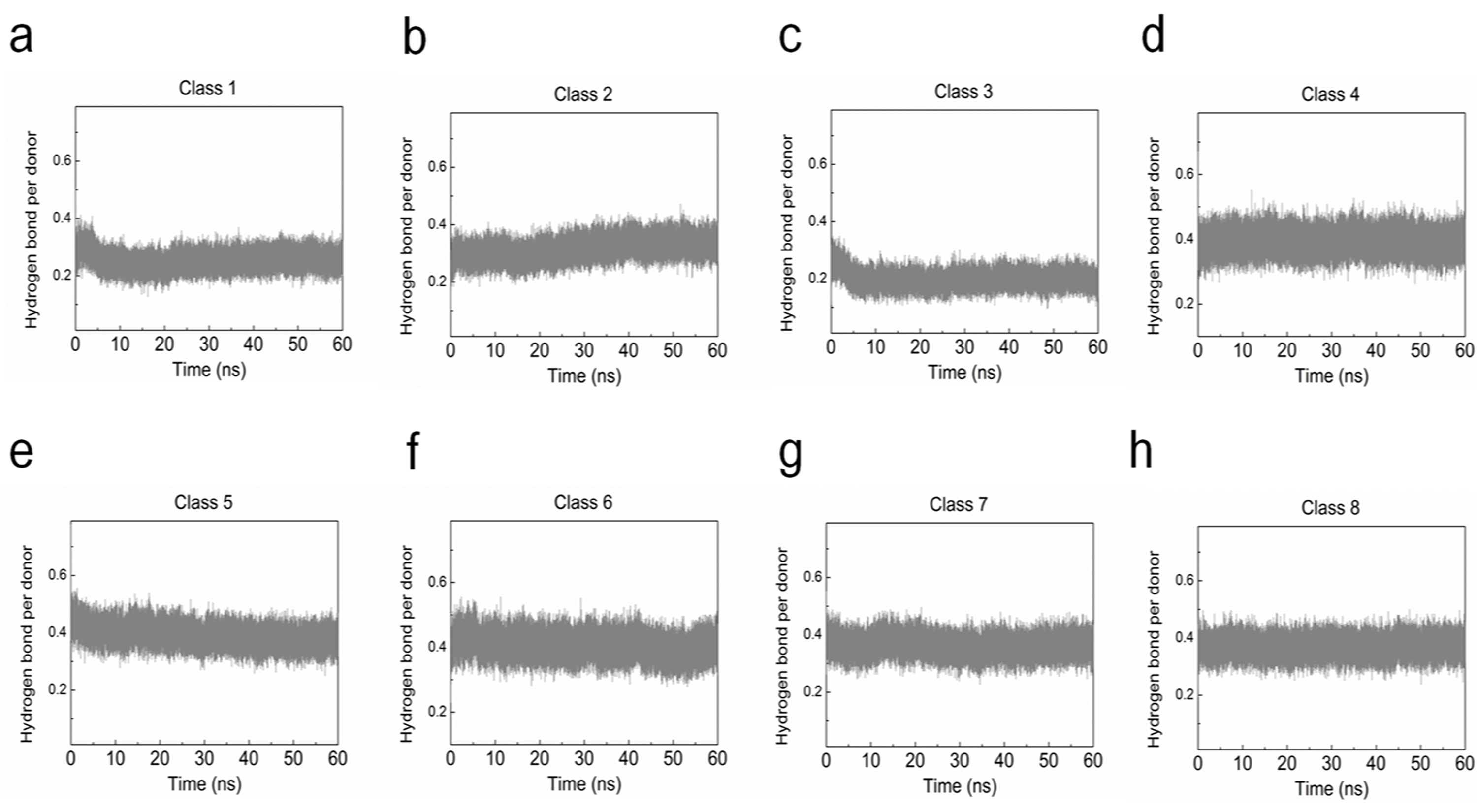

Supplement: Figure S2 — H-bonds per residue for polymorphic hIAPP fibrils. (TIF) [file pone.0088502.s002.tif]

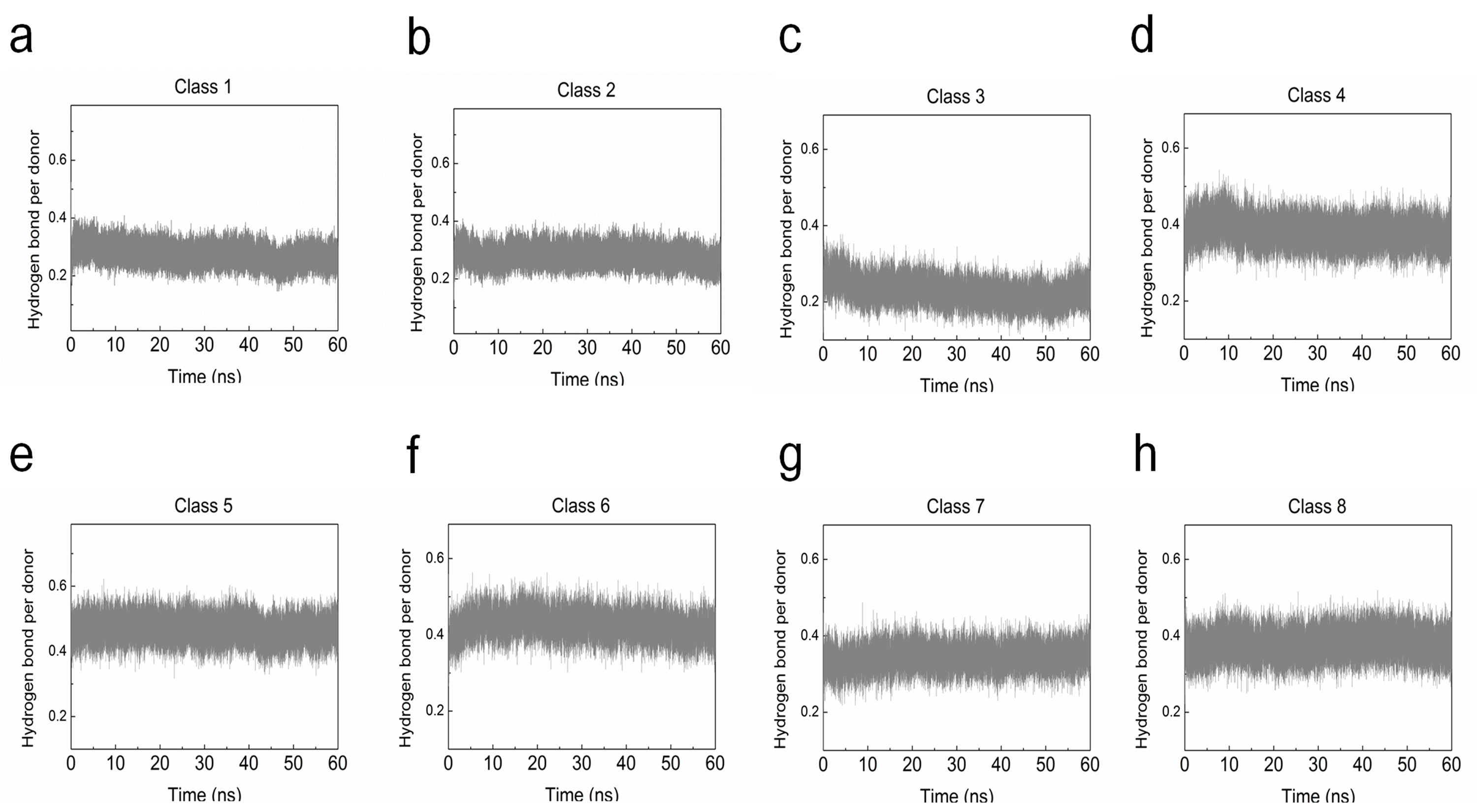

Supplement: Figure S3 — H-bonds per residue for mutated IAPP fibrils with their polymorphic structures. (TIF) [file pone.0088502.s003.tif]

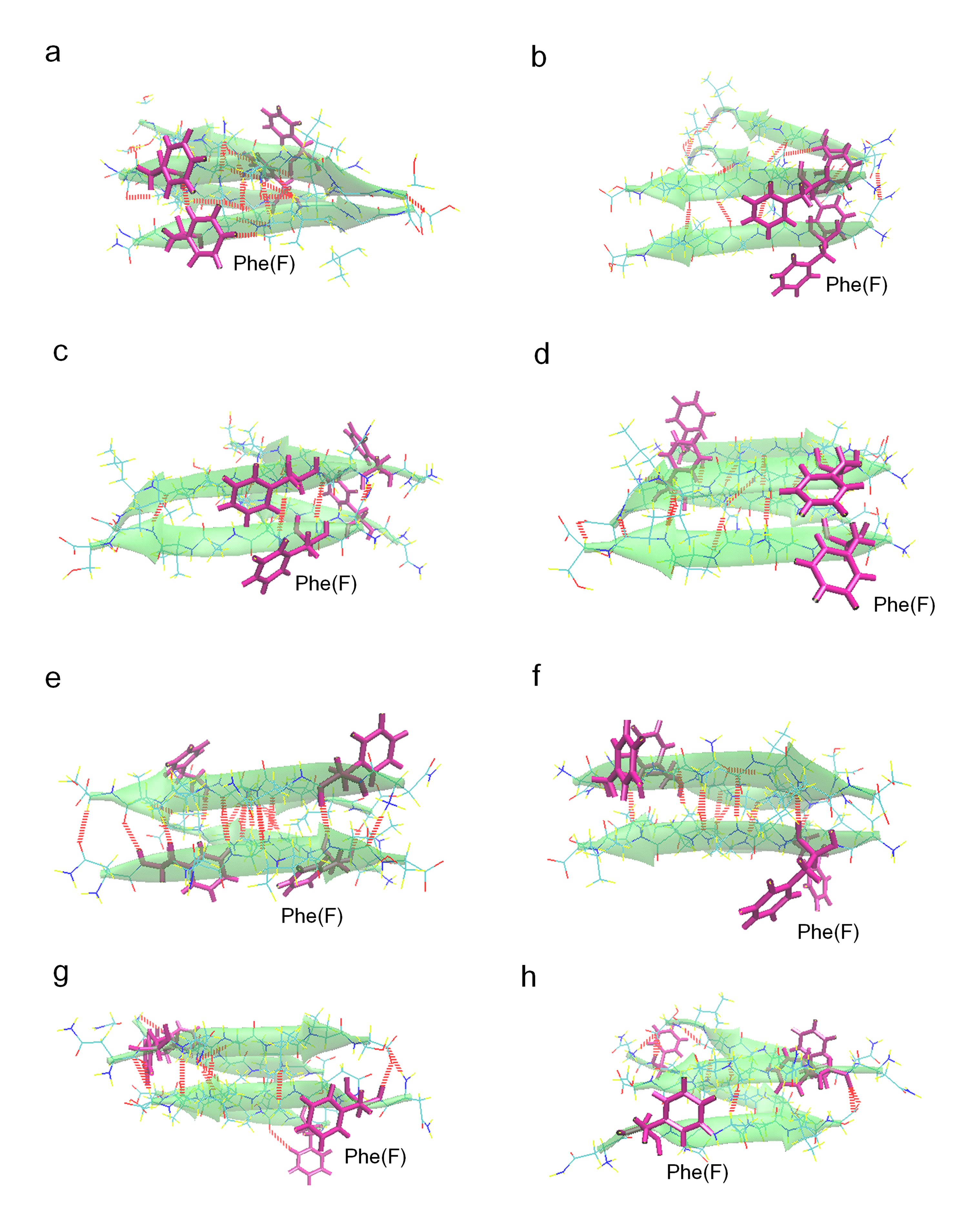

Supplement: Figure S4 — H-bond network for wild type fibril. Here, hydrogen bonds are indicated by red dotted lines, while hydrogen, oxygen, nitrogen, and carbon atoms are colored in yellow, red, blue, and cyan, respectively. (TIF) [file pone.0088502.s004.tif]

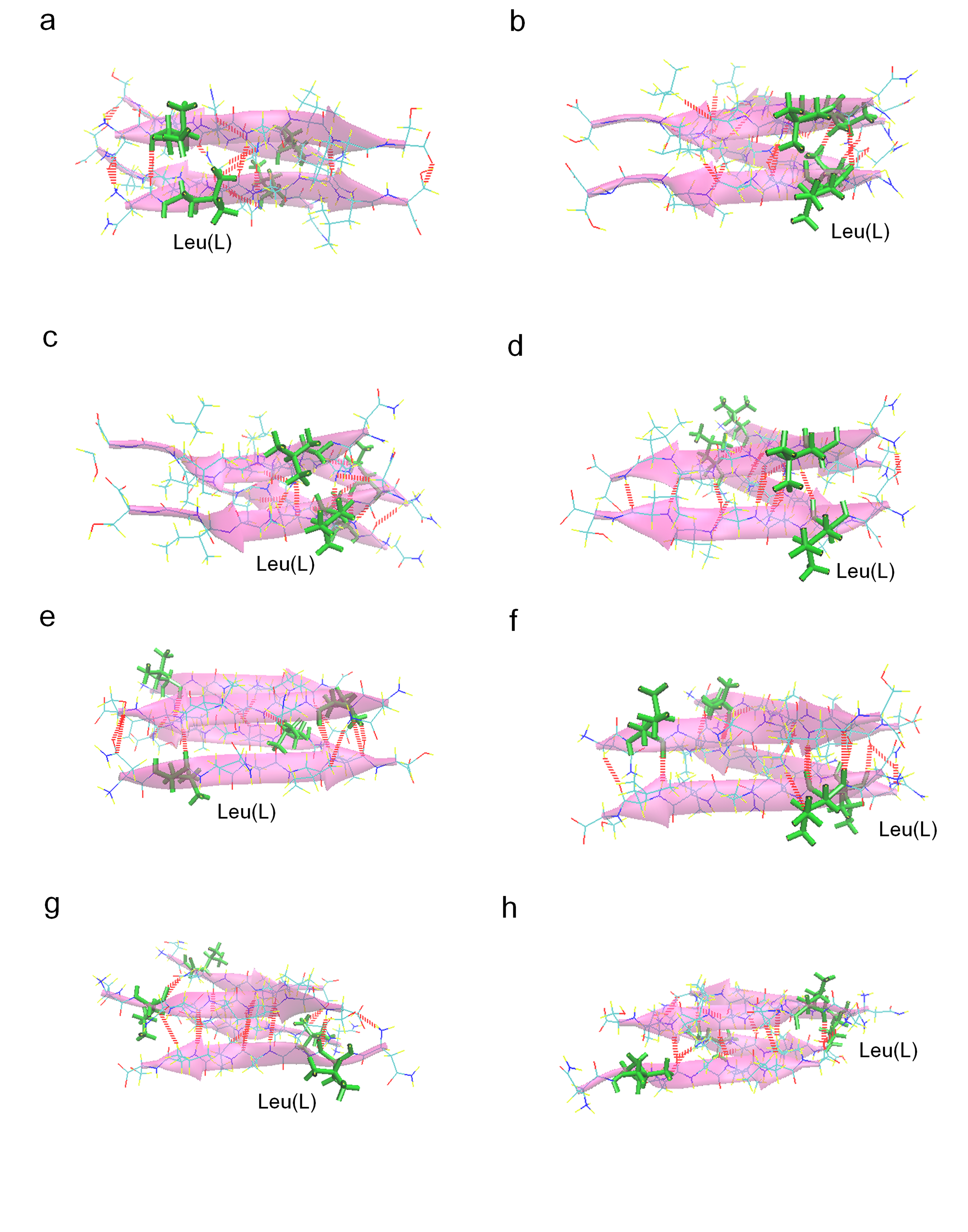

Supplement: Figure S5 — H-bond network for mutated fibril. (TIF) [file pone.0088502.s005.tif]

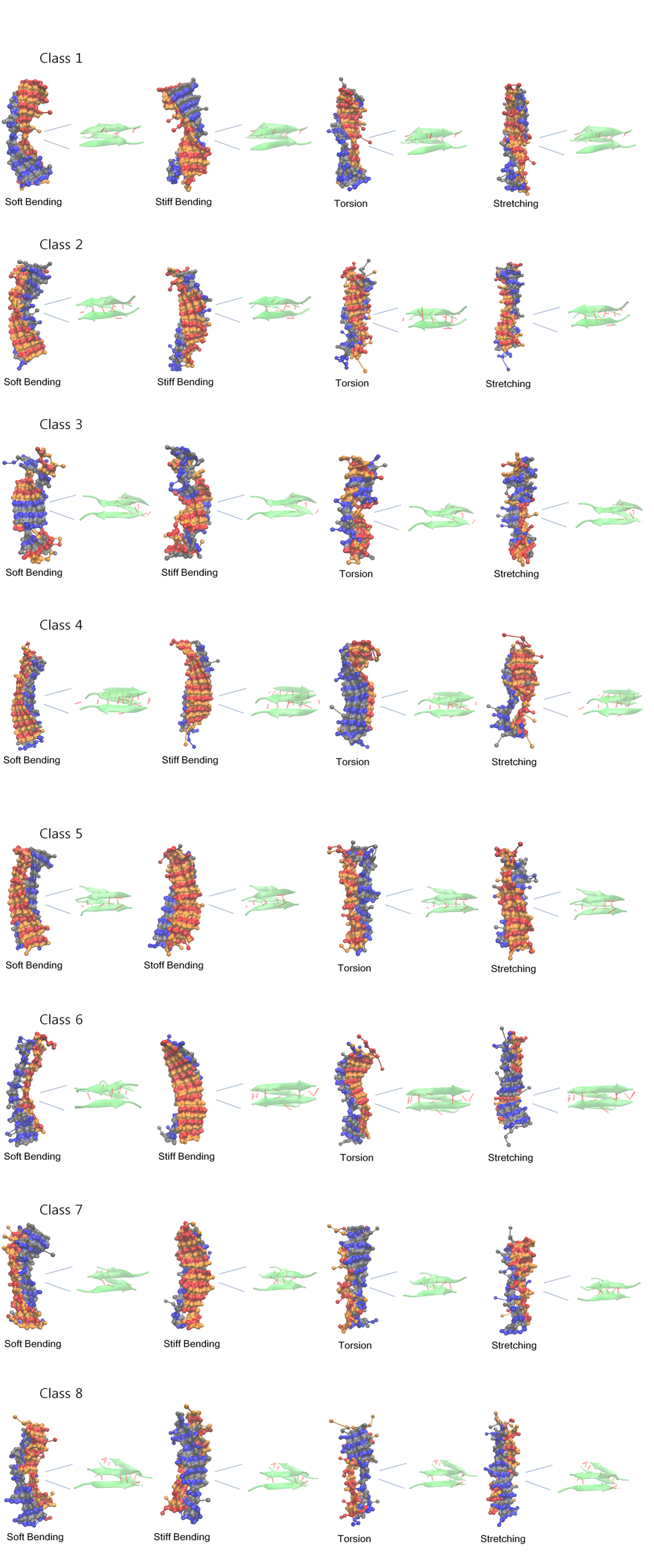

Supplement: Figure S6 — Hydrogen bond network for wild type fibrils that undergo deformation modes. (TIF) [file pone.0088502.s006.tif]

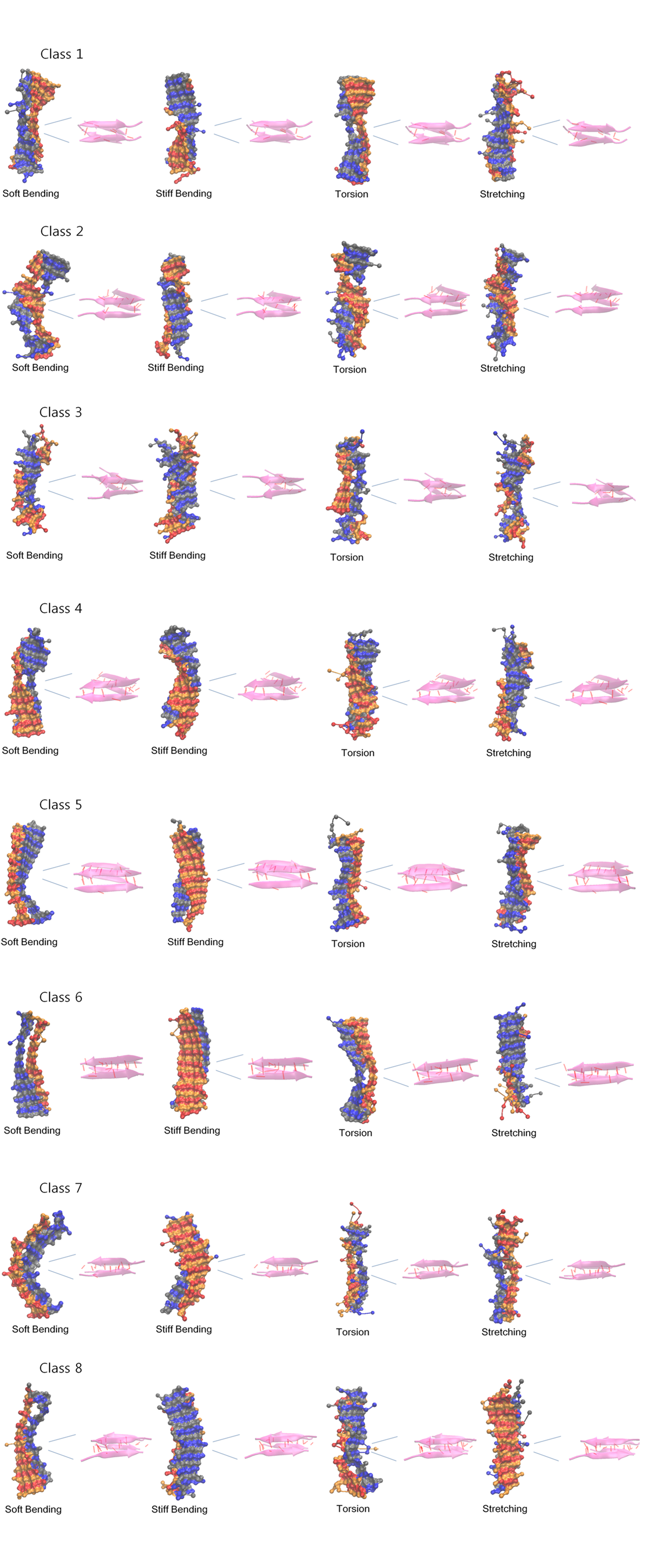

Supplement: Figure S7 — Hydrogen bond networks for mutated fibrils undergoing deformation modes. (TIF) [file pone.0088502.s007.tif]
