# Supplementary material for: Role of Sequence and Structural Polymorphism on the Mechanical Properties of Amyloid Fibrils
Source: PLoS One. 2014 Feb 14;9(2):e88502. doi: 10.1371/journal.pone.0088502 (PMC3925137; doi:10.1371/journal.pone.0088502)
Supplement: Methods S1 — Detailed supporting methods for the model of an amyloid fibril, equilibrium molecular dynamic simulation, and characterization of equilibrium dynamics of fluctuating amyloid fibrils. (DOCX) [file pone.0088502.s008.docx]

**METHODS S1**

**Role of Sequence and Structural Polymorphism on the Mechanical Properties of Amyloid Fibrils**

Gwonchan Yoon^1,2,#^, Myeongsang Lee^1,#^, Jae In Kim^1^, Sungsoo Na^1,*^, and Kilho Eom^3,*^

^1^Department of Mechanical Engineering, Korea University, Seoul, Republic of Korea

^2^Department of Mechanical Engineering, Boston University, Boston, Massachusetts, United States of America

^3^Biomechanics Laboratory, College of Sport Science, Sungkyunkwan University, Suwon, Republic of Korea

^#^These authors (G.Y. and M.L.) made equal contribution to this work.

*Correspondence should be addressed to K.E. (E-mail: [kilhoeom@skku.edu](mailto:kilhoeom@skku.edu)) or S.N. (E-mail: [nass@korea.ac.kr](mailto:nass@korea.ac.kr)).

**A. Construction of IAPP Fibril**

To construct the molecular structure of hIAPP fibril, we utilized the experimentally observed structure of hIAPP_20-29_ available in protein data bank (pdb) with pdb code of 2KIB [[1](#_ENREF_1)]. The sequence of hIAPP_20-29_ is given as “SNNFGAILS”. The hIAPP_20-29_ plays a role in the formation of amyloidogenic core that acts as a catalyst to assist the self-assembly to form hIAPP fibril. Based on the structure of hIAPP_20-29_, we have developed eight types of steric zipper patterns as described in a literature [[2](#_ENREF_2)]. In order to study the effect of genetic mutation on the mechanical properties of mutated IAPP fibril, we have taken into account the point mutation by substituting the sequence of phenylalanine (“F”) with leucine (“L”), since the sequence of rIAPP that does not lead to the expression of type II diabetes in a rat is given by “SNNLGAILS” [[3](#_ENREF_3)]. The mutation of hIAPP_20-29_ was implemented using visual molecular dynamics (VMD) package. The equilibrium conformations of hIAPP fibrils and rIAPP fibrils are obtained from equilibrium MD simulation as described in Section S1.2.

**B. Equilibrium Molecular Dynamics Simulation**

We carried out all-atom explicit water MD simulation in order to simulate the thermal fluctuation behavior of amyloid fibrils using NAMD package [[4](#_ENREF_4)] along with CHARMM27 force field [[5](#_ENREF_5)]. In this work, the equilibrium dynamics simulation of amyloid fibrils was conducted under explicit solvent condition using TIP3P water box solvent. The solvent box was made in such a way that the distance between solvent box and the outer atom of amyloid fibril is given as 15 Å. Before implementing the equilibrium dynamics simulation, we have performed the energy minimization process using conjugate gradient method with 10^4^ iteration steps. Subsequently, the equilibrium dynamics simulation was performed based on NVT ensemble at temperature of 300 K with time step of 2 fs. The trajectory of atoms constituting amyloid fibril is recorded for every 3 ps.

**C. Characterization of Equilibrium Conformations for Amyloid Fibril**

In order to describe the thermal fluctuation of the molecular structure of amyloid fibril, we have considered the trajectory of Cα atoms that constitutes the backbone of amyloid fibril at every 3 ps. The root-mean-square distance (RMSD) of an amyloid fibril is defined as

$RMSD\left(t\right)=\sqrt{\frac{1}{N}\sum_{j=1}^{N} \left(\mathbf{r}_{j}\left(t\right)-\mathbf{r}_{j}\left(0\right)\right)^{2}}$ (S1)

where **r***_j_* is the coordinate of the *j*-th Cα atom, *N* is the total number of Cα atoms constituting amyloid fibril, and 0 indicates the initial state (i.e. *t* = 0).

The bending angle between two adjacent β sheet layers is computed as follows.

$\theta=\cos^{-1}\left(\frac{\mathbf{n}_{k}\cdot\mathbf{n}_{k+1}}{\left\|\mathbf{n}_{k}\cdot\mathbf{n}_{k+1}\right\|}\right)$ (S2)

Here, **n***_k_* is the normal vector of a plane formed by *k*-th β sheet layer. The twist angle between two adjacent β sheet layers is defined as

(S3)

where **d***_k_* is the end-to-end distance vector between two adjacent β strands within the *k*-th β sheet layer.

**D. Quasi-Harmonic Analysis: Measurement of Natural Frequencies of Amyloid Fibril**

To characterize the vibrational behavior of amyloid fibril, we have considered principal component analysis (PCA) [[6-8](#_ENREF_6)] that allows for the estimation of the natural frequencies and their corresponding normal modes for amyloid fibril. The fluctuation matrix **Q** for amyloid fibril is defined as

$\mathbf{Q}=\left\langle\left\{\mathbf{r}\left(t\right)-\left\langle\mathbf{r}\left(t\right)\right\rangle\right\}\otimes\left\{\mathbf{r}\left(t\right)-\left\langle\mathbf{r}\left(t\right)\right\rangle\right\}\right\rangle$ (S4)

where **r**(*t*) is the atomic coordinates of *N* number of Cα atoms constituting the backbone of an amyloid fibril, a symbol indicates the tensor product, and an angle bracket < > represents an ensemble average. Here, it is noted that fluctuation matrix **Q** is the 3*N* × 3*N* matrix. Here, it should be noted that the root-mean-square fluctuation (RMSF) is given by

$RMSF=\sqrt{\frac{1}{N}\sum_{j=1}^{N} \left\langle\left|\mathbf{r}_{j}\left(t\right)-\left\langle\mathbf{r}_{j}\left(t\right)\right\rangle\right|^{2}\right\rangle}=\sqrt{\frac{1}{N}Tr\mathbf{Q}}$ (S5)

In order to compute the natural frequencies of amyloid fibril, we have utilized the spectral decomposition method [[9](#_ENREF_9)] that allows the representation of fluctuation matrix **Q** in the normal mode space as follows.

$\mathbf{Q}=\mathbf{V}\Xi\mathbf{V}^{T}=\sum_{k=1}^{3N-6} \xi_{k}\mathbf{v}_{k}\otimes\mathbf{v}_{k}$ (S6)

Here, Ξ is a diagonal matrix whose component is an eigenvalue for fluctuation matrix **Q**, **V** is the modal matrix whose column vector represents the normal mode vector, *ξ_k_* is the *k*-th eigenvalue of fluctuation matrix **Q**, and **v***_k_* is the *k*-th normal mode vector (i.e. *k*-th column vector of modal matrix **V**). Here, it should be noted that first six zero normal modes corresponding to the rigid body motions of amyloid fibril are excluded in the summation given in Eq. (S5). In this work, the spectral decomposition has been implemented based on component mode synthesis as delineated in our previous study [[10](#_ENREF_10)].

To relate the fluctuation behavior depicted by fluctuation matrix **Q** to vibrational characteristics that can be determined from stiffness matrix **K**, we employed the statistical mechanics theory [[11](#_ENREF_11)], which shows the relationship between two matrices **K** and **Q** as follows.

$\mathbf{K}=k_{B}T\sum_{j=1}^{3N-6} \xi_{j}^{-1}\mathbf{v}_{j}\otimes\mathbf{v}_{j}$ (S7)

where *k_B_* and *T* indicate the Boltzmann constant and absolute temperature, respectively. In order to obtain the natural frequencies of amyloid fibril, we have considered quasi-harmonic approximation [[12-14](#_ENREF_12)] that allows us to describe the equation of motion for fluctuating amyloid fibril in the form of

$\mathbf{M}\partial_{t}^{2}\mathbf{u}+\mathbf{Ku}=0$ (S8)

where **M** is the mass matrix of amyloid fibril, and **u** is the displacement vector of *N* number of Cα atoms constituting the backbone of amyloid fibril. Here, it should be noted that quasi-harmonic approximation depicted in Eq. (S8) is appropriate for understanding the low-frequency motion of protein molecules. For an amyloid fibril that vibrates, the displacement vector **u**(*t*) can be represented in the form of **u**(*t*) = **z**⋅exp[*iωt*], where *ω* is the natural frequency of amyloid fibril, **z** is the normal mode vector, and *i* is the unit of a complex number, i.e. $i=\sqrt{-1}$. Accordingly, the equation of motion given by Eq. (S8) becomes the eigenvalue problem as follows.

$\mathbf{Kz}=\omega^{2}\mathbf{Mz}$ (S9)

Based on Eq. (S8) and Eq. (S6), the natural frequency of amyloid fibril for the *j*-th normal mode can be obtained as follows.

$\omega_{j}=\sqrt{\frac{k_{B}T}{\xi_{j}M_{C}}}$ (S10)

where *M_C_* is the molecular weight of Cα atom.

**REFERENCES**

1. Nielsen JT, Bjerring M, Jeppesen MD, Pedersen RO, Pedersen JM, et al. (2009) Unique identification of supramolecular structures in amyloid fibrils by solid-state NMR spectroscopy. Angew Chem Int Ed 48: 2118-2121.

2. Sawaya MR, Sambashivan S, Nelson R, Ivanova MI, Sievers SA, et al. (2007) Atomic structures of amyloid cross-b spines reveal varied steric zippers. Nature 447: 453-457.

3. Hollander PA, Levy P, Fineman MS, Maggs DG, Shen LZ, et al. (2003) Pramlintide as an adjunct to insulin therapy improves long-term glycemic and weight control in patients with type 2 diabetes: a 1-year randomized controlled trial. Diabetes Care 26: 784-790.

4. Phillips JC, Braun R, Wang W, Gumbart J, Tajkhorshid E, et al. (2005) Scalable molecular dynamics with NAMD. J Comput Chem 26: 1781-1802.

5. MacKerell AD, Banavali N, Foloppe N (2000) Development and current status of the CHARMM force field for nucleic acids. Biopolymers 56: 257-265.

6. Amadei A, Linssen ABM, Berendsen HJC (1993) Essential Dynamics of Proteins. Proteins: Struct Funct Genet 17: 412-425.

7. Kitao A, Go N (1999) Investigating protein dynamics in collective coordinate space. Curr Opin Struct Biol 9: 164-169.

8. Hayward S, Go N (1995) Collective Variable Description of Native Protein Dynamics. Annu Rev Phys Chem 46: 223-250.

9. Golub GH, Van Loan CF (1996) Matrix Computations. Baltimore: The Johns Hopkins University Press.

10. Kim J-I, Na S, Eom K (2009) Large Protein Dynamics Described by Hierarchical Component Mode Synthesis. J Chem Theor Comput 5: 1931-1939.

11. Weiner JH (1983) Statistical mechanics of elasticity: Dover publication.

12. Brooks B, Karplus M (1983) Harmonic Dynamics of Proteins: Normal Modes and Fluctuations in Bovine Pancreatic Trypsin Inhibitor. Proc Natl Acad Sci USA 80: 6571-6575.

13. Teeter MM, Case DA (1990) Harmonic and quasiharmonic descriptions of crambin. J Phys Chem 94: 8091-8097.

14. Janezic D, Venable RM, Brooks BR (1995) Harmonic-Analysis of Large Systems .3. Comparison with Molecular-Dynamics. J Comput Chem 16: 1554-1566.
